# Gold Nanoparticle Aggregation as a Probe of Antifreeze (Glyco) Protein-Inspired Ice Recrystallization Inhibition and Identification of New IRI Active Macromolecules

**DOI:** 10.1038/srep15716

**Published:** 2015-10-26

**Authors:** Daniel E. Mitchell, Thomas Congdon, Alison Rodger, Matthew I. Gibson

**Affiliations:** 1 Department of Chemistry, University of Warwick, Gibbet Hill Road, Coventry, CV4 7AL, UK; 2 MOAC DTC, University of Warwick, Gibbet Hill Road, Coventry, CV4 7AL, UK

## Abstract

Antifreeze (glyco)proteins are found in polar fish species and act to slow the rate of growth of ice crystals; a property known as ice recrystallization inhibition. The ability to slow ice growth is of huge technological importance especially in the cryopreservation of donor cells and tissue, but native antifreeze proteins are often not suitable, nor easily available. Therefore, the search for new materials that mimic this function is important, but currently limited by the low-throughout assays associated with the antifreeze properties. Here 30 nm gold nanoparticles are demonstrated to be useful colorimetric probes for ice recrystallization inhibition, giving a visible optical response and is compatible with 96 well plates for high-throughout studies. This method is faster, requires less infrastructure, and has easier interpretation than the currently used ‘splat’ methods. Using this method, a series of serum proteins were identified to have weak, but specific ice recrystallization inhibition activity, which was removed upon denaturation. It is hoped that high-throughput tools such as this will accelerate the discovery of new antifreeze mimics.

To survive in the harsh cold environments at high altitude or in the Earth’s polar regions, Nature has evolved a series of mechanisms to enable extremophiles to survive, and thrive. One particular method of survival employed by freeze-avoiding^1^ (as opposed to freeze tolerant)^2^ species is the production of antifreeze proteins and antifreeze glycoproteins (AF(G)Ps)^3^. These proteins act to i) lower the equilibrium freezing point in a non-colligative fashion – thermal hysteresis^4^ (TH); ii) shape ice crystals (via binding to specific crystallographic faces) –dynamic ice shaping (DIS); iii) inhibit the growth of ice crystals – ice recrystallization inhibition (IRI)^5^. The property of IRI is particularly interesting, as ice crystal growth during thawing has been associated with cellular damage during cryopreservation^6^. Considering this, AF(G)Ps have been investigated for their ability to enhance cryopreservation^6^, with the aim of improving the availability of transplantable materials for regenerative medicine. The results of this have been mixed, with reports of AF(G)Ps both enhancing and reducing cell viability post-thawing^6^,^7^,^8^. This has been attributed to their unwanted effects of DIS/TH, which can promote the formation of needle-like (spicular) ice crystals^9^. There are also concerns about the toxicological and immunological affects of AF(G)PS^10^, along with the inherent cost of producing/purifying them from natural sources.

Therefore, the development of new IRI-specific molecules/macromolecules has been pursued. Ben *et al.* have reported that simplified glycopeptides and glycolipids have potent IRI and can enhance cryopreservation^11^,^12^,^13^. Gibson and co-workers have shown that synthetic polymers can mimic AF(G)Ps function and shown this to enhance red blood cell cryopreservation^14^,^15^,^16^. However, there are still few examples of synthetic AF(G)P mimics, which is in part limited by the time-consuming nature of the assays required to probe their activity. The TH assay requires the growth of single ice crystals in nanolitre droplets, whereas IRI activity is tested by the ‘splat’ assay; this involves forming many wafers of sub 10 micron ice crystals and monitoring their growth over time^17^. Neither of these assays are suitable for high-throughput analysis, requiring temperature-controlled microscope stages and significant image analysis. To speed the process Ben *et al.* introduced domain recognition software^18^, which aids image processing but the data collection burden is not removed. Several other methods involving capillaries and light scattering have been suggested^19^,^20^,^21^, but these do not give quantitative data or require users ‘interpretation’ of the results and have not been widely adopted.

Interestingly, Kim *et al.*^22^, suggested that gold nanoparticles could be used to measure thermal hysteresis colourimetrically. Gold nanoparticles show a distinct shift from red to blue colouration upon aggregation and has been employed for a range of bioassays from DNA-sequence comparison^23^, bacteria analysis^24^, viral analysis^25^, glycan analysis^26^, metal detection^27^ and more. Kim *et al.*^22^ reported that the aggregation of gold particles correlated with the TH activity of an AFP. The mechanism of this was not clear though as freezing then thawing gold nanoparticles should not actually be affected by TH. The macroscopic effect of TH is to lower the freezing point (by circa. 2 °C) but in the reported assays, activity was seen at temperatures significantly below this and using AFP concentrations which are too low for TH activity. We believe these observations actually correlates better with IRI activity, which is maintained at lower concentrations^28^,^29^. When an aqueous solution is frozen, the ice crystals will grow larger by ice recrystallization, reducing the overall ice surface area. As all solutes are excluded from ice crystal, the gold particles in between will be forced closer together leading to aggregation. Anything that possesses IRI activity would prevent this ice crystal growth, meaning the overall surface area would be larger preventing aggregation from occurring. Despite the previous reports suggesting this is a TH assay, if it can be adapted for IRI the method is very appealing as it could be conducted in multiwell plates, the output can be read by a standard plate-reader (or visually) and only needs a standard −20 °C freezer. The combination of readily available equipment requirements and multi-well plate compatibility would make this this a potential high-throughput assay for testing a diverse range of compounds.

In this manuscript, we report a study into the use of gold nanoparticles (AuNPs) as a simple, and rapid, tool for screening for IRI activity. Using well-defined synthetic polymers, with known IRI activity, but no (or weak) thermal hysteresis we show that the inhibition of gold particle aggregation correlates with IRI. The utility of this in a 96 well plate format is reported to enable higher-throughput screening than compared to the splat assay.

## Results and Discussion

**Figure 1:**
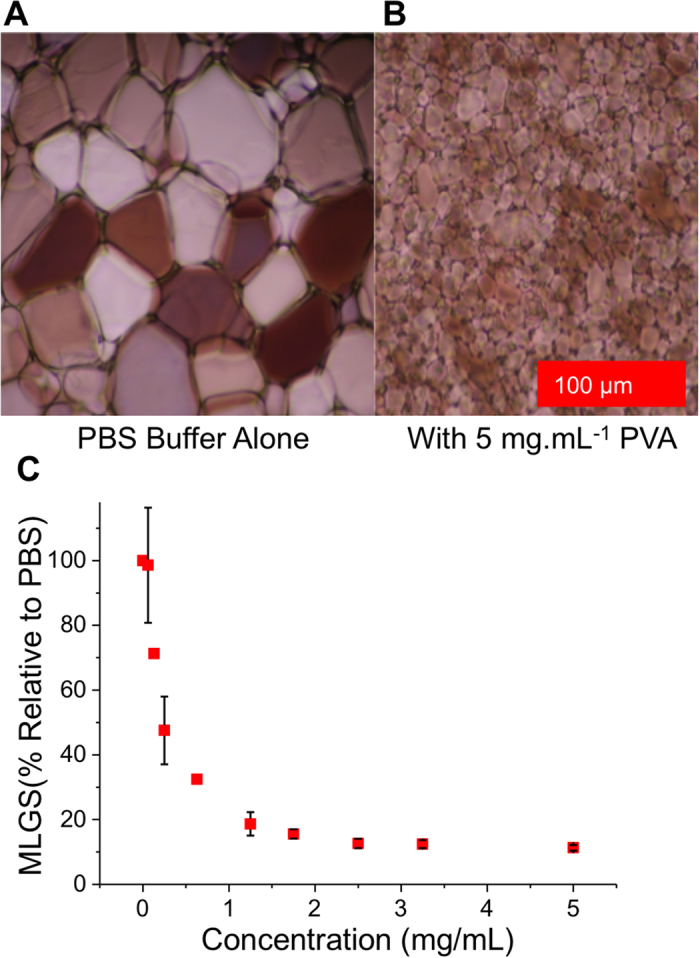
Ice recrystallization inhibition activity of PVA; (A) Example micrograph of crystals grown in PBS alone; (B) Example micrograph of crystals grown in 5 mg.mL^−1^ PVA; (C) IRI activity as a function of polymer concentration. MLGS = mean largest grain size relative to a PBS control (%). Error bars represent the standard deviation from at least three measurements. (PVA MW = 6.8 kDa).

To test the applicability, if any, of the reversible freezing of colloidal gold as a probe for IRI activity (rather than the previously explored thermal hysteresis)^22^ a range of synthetic polymers with known IRI activity were selected. Poly(vinyl alcohol), PVA, was chosen as the positive control due to is well-known IRI activity, which has been investigated in detail^28^. As a negative control poly(ethylene glycol) and poly(*N*-vinyl pyrolidone), PVP, were selected as they have no significant IRI in the relevant concentration range^30^,^31^. Figure 1 shows the results of a ‘splat’ test, which measures the ripening of ice crystals over time, in both the absence and presence of PVA, highlighting its inhibitory effect on ice crystal growth. Briefly, this assay involves seeding large numbers of small ice crystals and monitoring their growth to obtain the mean largest grain size (MLGS) to quantify activity. Using this assay, it has been shown that concentrations of PVA below 1 mg.mL^−1^ are sufficient to halt ice crystal growth.

**Figure 2:**
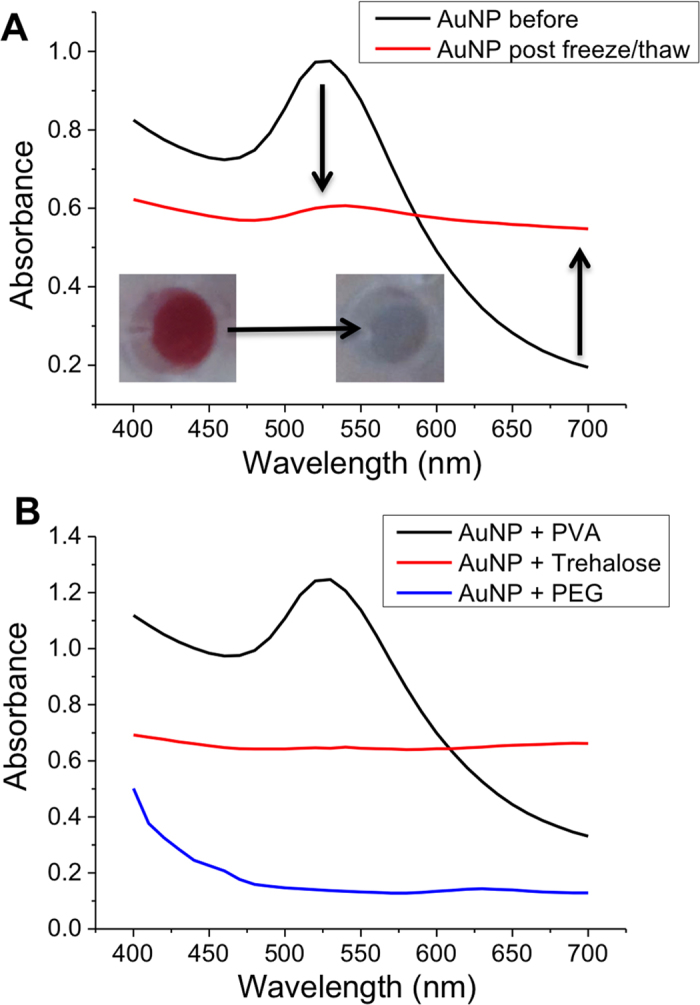
Absorbance spectra changes of gold nanoparticles (AuNPs) solutions upon freeze/thawing. (**A**) Effect of freezing and thawing gold nanoparticle solutions, with decrease in absorbance at 520 nm. Inset photos show colour changes associated with AuNP freeze/thaw; (**B**) Gold nanoparticle UV-Vis spectra after freeze-thaw in the presence of additives at a concentration of 10 mg.ml^−1^ (PEG MW 4 kDa, PVA MW 6.8kDa). Arrows are to guide the eye in the direction of change.

30 nm mercaptosuccinic acid (MSA) functionalized gold particles prepared using literature methods^22^,^32^. The gold nanoparticle solutions containing the compounds to be tested for IRI were frozen in 96-well plates at −20 °C for 2 hours in a standard laboratory freezer (−20 °C). The samples were then thawed slowly at 23 °C (ambient temperature) to maximise ice recrystallization, and their UV-Visible absorption spectra recorded as well as optical photographs, as shown in Fig. 2A. From visual inspection of the samples it was clear that the AuNPs with added PVA were still dispersed (characteristic red colour), but other samples had aggregated. The concentration dependence of AuNPs with PVA was also measured and the success of the assay was found to not depend on gold concentration. A concentration of ~80 ug.mL^−1^ is used from this point onwards (Supp. Info).

**Figure 3:**
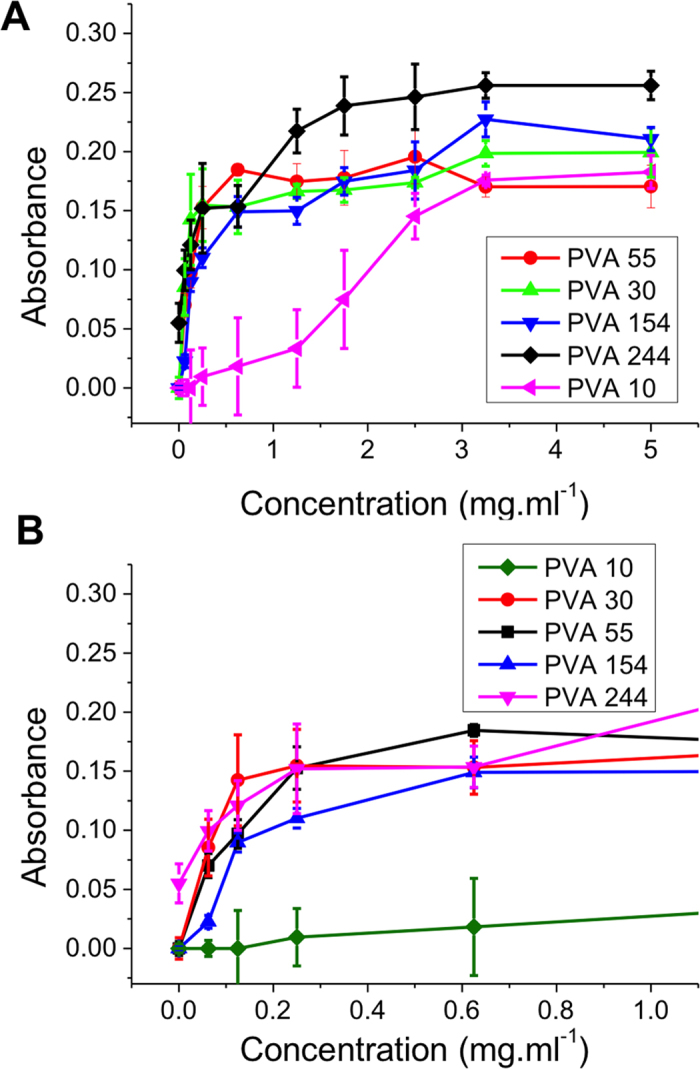
Concentration dependence on the absorbance at 520 for different degrees of polymerization of PVA post freeze/thaw. (**A**) Concentration dependence of IRI activity from 0 to 5 mg.ml^−1^; (**B**) Zoomed-in region from 0 to 1 mg.ml^−1^ showing concentration dependence resolution for low concentrations. Error bars represent ± SD from a minimum of 3 repeats.

In order to ‘score’ the relative degree of aggregation post-thawing, the reduction in the absorbance peaks at 520 nm between pre- and post-thaw were measured to give a qualitative comparison between different additives (see Supporting Information) with higher values indicating less aggregation and hence more IRI activity. To assess the use of this colloidal assay a range of different molecular weight PVAs from 800–20000 g.mol^−1^ were synthesised using RAFT/MADIX polymerization^28^ (Supp. info). The PVA’s are labelled according to their degree of polymerization (chain length) from this point. Above 1 mg.mL^−1^ all the PVAs prevented aggregation of the gold particles after a freeze/thaw cycle, in agreement with the ‘splat’ assay. When the concentration was decreased below 1 mg.mL^−1^ there were clear differences between the polymers of different molecular weight, as shown in the zoomed-in region in Fig. 3. The largest polymer (PVA_244_) was capable of inhibiting aggregation as low as 0.1 mg.mL^−1^, but the shortest (PVA_10_) lead to AuNP aggregation in this concentration range, demonstrating that high molecular weight polymers have increased IRI activity.

**Figure 4:**
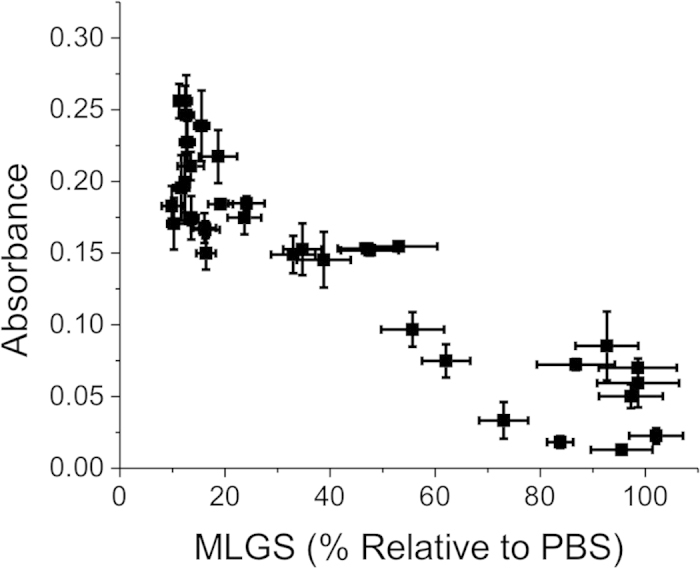
Comparison of MLGS values (relative to a PBS control) versus absorbance from all PVA/AuNP aggregation assay. MLGS is expressed as a percentage of PBS buffer control.

In an attempt to correlate these observable spectral features with our ‘gold standard’ splat assay, the whole data set was plotted as Abs_520_ versus MLGS, Fig. 4. There was a clear correlation between the MLGS and Abs_520_, with lower absorbance values correlating with smaller MLGS values. Importantly, there were some outliers, giving rise to false positives in some cases. This highlights the potential as a screening tool, but still needs the complimentary splat test to confirm any findings. We believe these outliers are related to the relatively high concentration of the low molecular weight PVA’s which correlate to these data points.

Variable volume experiments were also conducted to ensure that this was a recrystallization-specific effect. Briefly, smaller volumes will thaw faster, reducing the opportunities for recrystallization, compared to larger volumes. A short screen of this (Supp. Info.) confirmed that 100 μL total volume per well (in a 96 well plate) lead to aggregation of the gold after freeze-thaw, but lower volumes lead to less aggregation. This also supports our hypothesis that recrystallization inhibition, not thermal hysteresis is the effect being probed and that the conducting these experiments in 96 well plates, as opposed to 384 well plates may be preferable.

**Figure 5:**
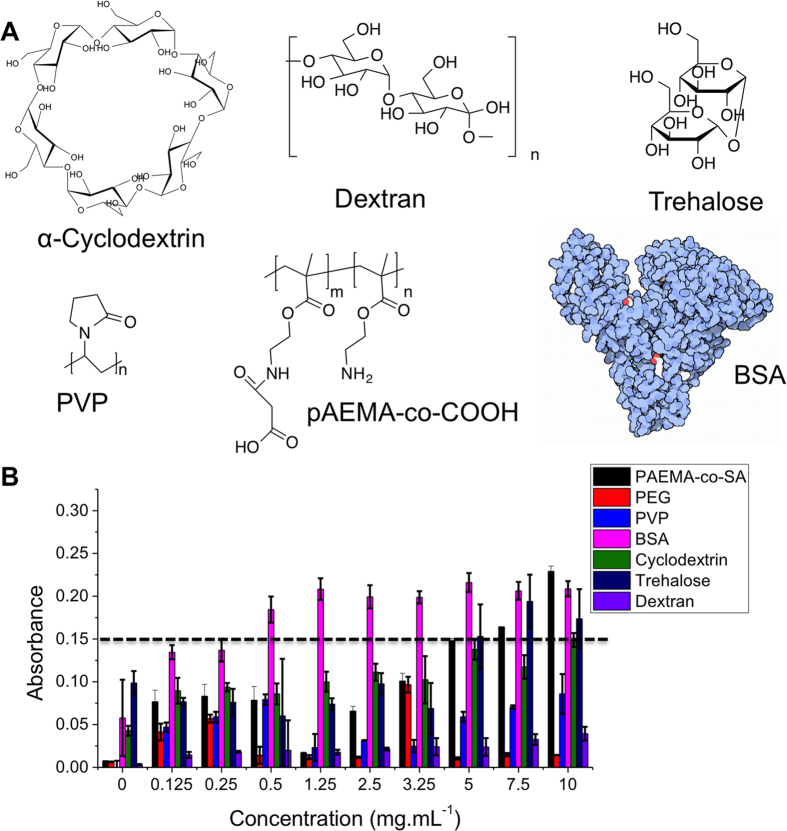
Screening for IRI activity using AuNP Assay; (A) Chemical structures of the compounds being interrogated; (B) AuNP colloidal aggregation results. Error bars represent ± SD from a minimum of 3 repeats. Dotted line is to guide the eye – data points above this indicate IRI activity.

The data presented above suggests that the AuNP method may be a useful screening assay for IRI activity. To probe this, a range of compounds with and without known IRI activity were screened; Poly(amino-ethyl methacrylate) 50% carboxylated with succinic anhydride, PAEMA-*co*-SA (35 kDa), α-cyclodextrin dextran (40 kDa), trehalose, poly(vinyl pyrolidone) (PVP, MW 40 kDa) and bovine serum albumin (BSA). Of these only PAEMA-*co*-SA is known to have IRI activity and this is significantly less active than PVA, typically requiring concentrations of ~20 mg.mL^−1^ to have IRI activity, compared to 1 mg.mL^−1^ for PVA^33^. The IRI activity of trehalose and other sugars has been reported. However, this required concentrations of 220 mM (which is >50 mg.mL^−1^) to show significant ice inhibition and is only considered to be weakly active, with close to no activity in the concentration range used here^34^. PVP is a well known kinetic hydrate inhibitor, but does not have strong IRI^35^. Figure 5 shows the results of screening 6 compounds/macromolecules for IRI using this AuNP based assay.

**Figure 6:**
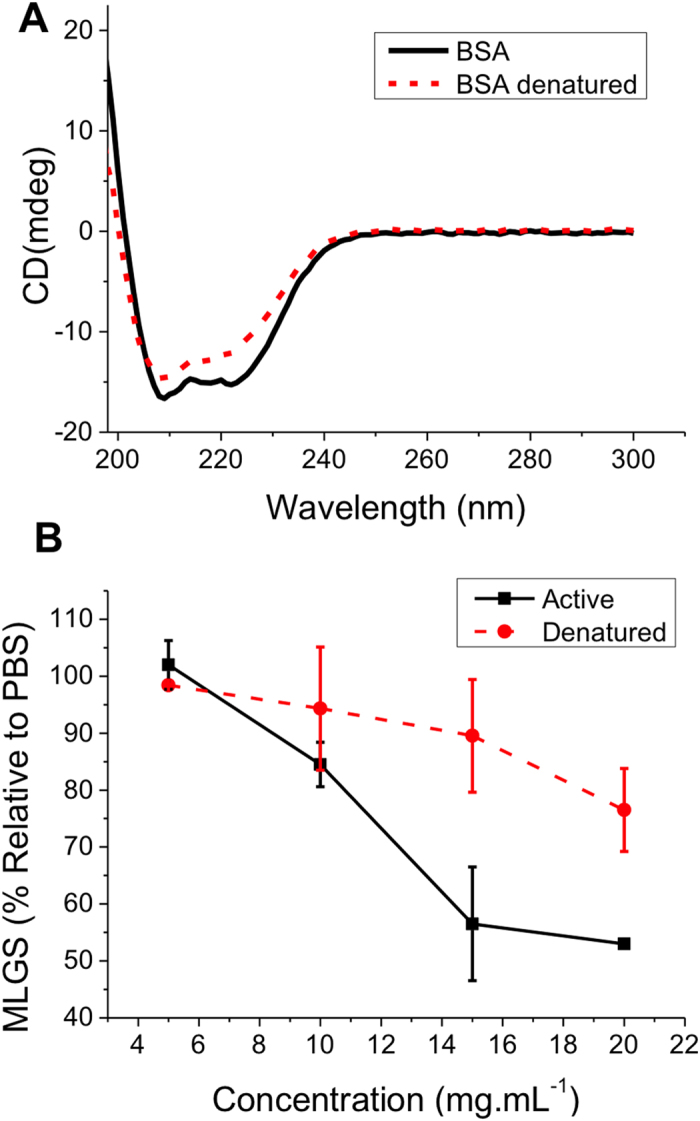
IRI activity of BSA. (**A**) Circular dichroism spectra of BSA at 500 μg.mL^−1^ before and after heat-denaturation; (**B**) Comparison of active and denatured BSA. Error bars represent ± SD from a minimum of 3 repeats. MLGS = mean largest grain size relative to PBS control.

As expected, PAEMA-*co*-SA showed a reduction in aggregation across a range of concentrations, in line with its known IRI activity, which correlates well with its MLGS (Supp. Info). Using dye inclusion assays PAEMA-*co*-SA shows no activity, suggesting that it is not acting as a surfactant, which could provide stabilisation to the AuNPs and is a potential source of false-positives^30^,^33^. PVP, dextran and cyclodextrin solutions all aggregated following freeze/thaw for all concentrations tested. This is in agreement with observations that none of these have IRI activity in the splat test. Most interestingly, BSA gave a result that would indicate IRI activity. This could be interpreted as it stabilising the gold nanoparticle surface (through non-specific absorption) or a real IRI affect. To the best of our knowledge, the IRI activity of BSA has not been quantitatively studied so we set out to investigate this. To ensure that we were probing protein-structure related effects, some of the BSA was denatured by heating (95 °C for 30 minutes). Circular dichroism spectroscopy (Fig. 6A) confirmed this by the decrease in molar elipticity at 210 and 225 nm. These changes seen in the CD spectra were small, but BSA is known to be quite tolerant to heating and can refold, although not necessarily into the same conformation^36^,^37^. The native, and denatured forms were then subjected to the splat test as a function of concentration, Fig. 6B.

**Figure 7:**
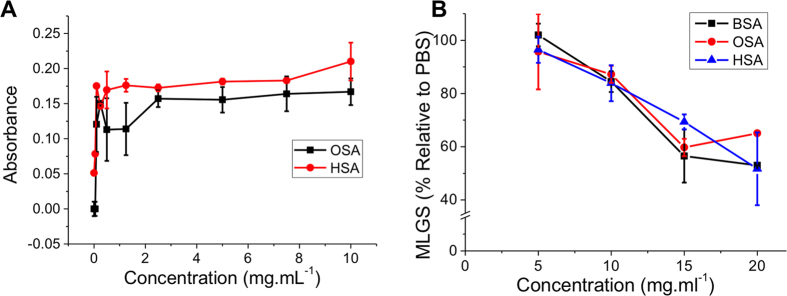
IRI activity of serum proteins. (**A**) Gold nanoparticle aggregation assay; (**B**) ‘Splat’ assay. BSA = bovine serum albumin, OSA = ovine (sheep) serum albumin and HSA = human serum albumin. MLGS = mean largest grain size relative to PBS control. Error bars indicate SD from a minimum of 3 repeats.

The splat test revealed that folded BSA does have real, if not very potent, IRI activity. This is surprising, as in our hands very few synthetic materials or proteins show any appreciable IRI with PAEMA-*co*-SA and PVA being amongst the few. The denatured BSA showed no IRI activity, indicating a remarkable tertiary-structure dependence, which has not been previously explored. This is of particular interest as many cryoprotectant solutions contain large quantities of serum proteins and this data implies that modulation of ice crystal growth may be an unexpected benefit in these systems. To determine whether this is a general property of serum proteins, human serum albumin (HSA) and ovine serum albumin (OSA) were also tested using both the AuNP and splat test methods. The AuNP screening method indicated some freeze-thaw resistance and the splat test confirmed that the OSA and HSA both had activity in line with the BSA, Fig. 7.

BSA is often used as a negative control in antifreeze protein assays (although at lower concentrations than used here) this should be taken into account in the future as it clearly does have activity, although the features that cause this are not clear. One role of serum proteins *in vivo* is as a carrier for various substances such as fatty acids and certain steroids, while also maintaining the oncotic pressure in the blood^38^,^39^. To carry these substances it possesses several binding domains and hydrophobic regions^40^. This distribution of hydrophobic and hydrophilic regions is a common feature in antifreeze proteins perhaps explaining the reason for its activity^41^. In a small molecule context, Ben *et al.* has shown that addition of increasingly hydrophobic alkyl chains to sugars can result in enhanced IRI which would seem to agree with this hypothesis for BSA activity^42^.

This study shows that AuNP reversible freeze/thaw cycles and the subsequent aggregation appears to be linked to the rate of ice crystal growth (recrystallization) rather than the previously hypothesized TH activity^22^. Our results demonstrate that it may be a useful tool for screening IRI, but that splat tests are still required to confirm activity of newly identified compounds. In particular, this method may be useful when screening structural variations of a single material (e.g. a polymer) where any nonspecific effects (such as particle binding) are common to all the structures, enabling false positives to be removed. The identification of serum albumin activity also suggests that IRI might be a more common property of proteins than previously thought. This opens up the opportunity to identify latent antifreeze-protein activity from non-extremophile organisms.

## Conclusions

Here the application of gold nanoparticles as convenient probes for ice recrystallization inhibition activity has been investigated. During thawing, extensive ice crystal growth occurs, reducing the available surface area for particles, meaning the gold particles are more likely to aggregate if crystal growth occurs. UV-visible spectroscopy can be employed to probe activity by measuring the characteristic red to blue shift of AuNP solutions as they aggregate. The degree of aggregation was found to correlate well with ice recrystallization inhibition activity determined by the (gold standard) splat assay. Previous studies had suggested that this reversible assay was due to freezing point depression (thermal hysteresis) but here synthetic polymers without freezing point depression activity gave strong aggregation inhibition, confirming IRI not TH was the mechanism being probed.

This assay has significant advantages in terms of speed and throughput compared to the splat test, which is the current ‘gold standard’ for ice recrystallization. This includes compatibility with 96 well plates for automated analysis and the use of a standard −20 °C lab freezer and easy colourimetric readout. Using this method, the IRI activity of serum proteins was identified for the first time and quantified by the ‘splat’ test and linked to the tertiary structure/folding of the proteins. At this time, the potential for false positives cannot be ruled out across all chemical space, but this represents a step towards a fully automated IRI activity screening platform.

## Experimental Section

### Physical and Analytical Methods

^1^H and ^13^C NMR spectra were recorded on Bruker DPX-300 and DPX-400 spectrometers using deuterated solvents purchased from Sigma-Aldrich. Chemical shifts are reported relative to residual non-deuterated solvent. Infrared data was recorded on a Bruker Vector 22 GI003097. The THF GPC system comprised of a Varian 390-LC-Multi detector suite fitted with differential refractive index (DRI), light scattering (LS) and ultra-violet (UV) detectors equipped with a guard column (Varian Polymer Laboratories PLGel 5 μm, 50 × 7.5 mm) and two mixed D columns of the same type. The mobile phase was THF with 5% triethylamine (TEA) eluent at a flow of 1.0 mL/min, and samples were calibrated against Varian Polymer Laboratories EasiVials linear poly(styrene) and poly(methylmethacrylate) standards (162–2.4 × 10^5^ g/mol) using Cirrus v3.3. Characterization of AuNPs was carried out using differential light scattering (DLS) spectroscopy (Malvern Instruments Zetasizer Nano-ZS). Circular Dichroism (CD) spectra were recorded on a spectropolarimeter (Jasco J-720, Jasco UK) using a data interval of 0.2 nm. Samples were dissolved in PBS buffer diluted two-fold with deionised water, and the spectrum was measured 16 times and averaged. The spectrum of a blank sample containing only buffer was then subtracted giving a final spectrum for each protein. UV-Visible spectroscopy was conducted on a microplate reader; Synergy HT multi-mode microplate reader, BioTek UK.

### Materials

Hydrogen tetrachloroaurate trihydrate (HAuCl_4_.3H_2_O), sodium citrate dehydrate (C_6_H_5_Na_3_O_7_.2H_2_O), mercaptosuccinic acid (MSA), dextran, α-cyclodextrin (MW 972.84 g.mol^−1^), trehalose, bovine serum albumin (BSA), human serum albumin (HSA), ovine serum albumin (OSA) and poly(ethylene glycol) (PEG) were purchased from Sigma Aldrich. Poly(amino ethyl methacrylate – co- succinic anhydride) was synthesized as previously reported^30^ and poly(vinyl alcohol) was synthesized as detailed in the ESI. All reagents were of analytical grade. Commercial polymers were dialysed against deionized water for 24 hours with 5 water changes prior to use against a 3000 MWCO membrane. Phosphate-buffered saline (PBS) solution was prepared using preformulated tablets (Sigma-Aldrich) in 200 mL of Milli-Q water (>18.2 Ω mean resistivity) to give [NaCl] = 0.138 M, [KCl] = 0.0027 M, and pH 7.4.

### Synthesis of Gold Nanoparticles

Gold Nanoparticle (AuNPs) were synthesized according to the literature^32^. Briefly, 6.7 mg of hydrogen tetrachloroaurate was dissolved in 50 mL of distilled water in a 50 mL round bottom flask, equipped with a magnetic stir bar. The solution was then heated to 100 °C while stirring and 2 mL of sodium citrate solution (2 mM, 17.6 mg) was added. The solution changed colour to purple and then red and the reaction was allowed to proceed for a further 20 minutes. The colloidal suspension was then allowed to cool to room temperature and 2 mL of mercaptosuccinic acid solution (30 mM, 9 mg) was added, and the functionalization was allowed to proceed overnight at room temperature. The resulting concentration of AuNPs was 58 μg.mL^−1^. Dynamic light scattering confirmed that the gold particles were ~30 nm in size.

### Freeze-Thaw Assay

The standard assay involved the addition of 50 μL of test product prepared as a serial dilution from 10–0 mg.mL^−1^ (or higher concentrations as needed) in a 96 well plate. To this, 50 μL of as-prepared AuNPs were added. To determine the affect of the solution volume on the assay the quantities of test and AuNP solution were varied although the ratio remained 1:1. All experiments were undertaken in triplicate. Freeze thaw was achieved by placing the plate in a standard domestic freezer for 2 hours at −20 °C and leaving to thaw on the lab bench at room temperature. The UV-vis spectra were corrected for scattering by drawing a straight line between 450 and 680 nm and measuring the absorbance at 520 nm, between this line and the peak. The optimal concentration of gold is 80 ug.mL^−1^ (see Supp. Info).

### Ice Recrystallization inhibition (splat) assay

Ice recrystallization inhibition was measured using a modified splay assay^17^. A 10 μL sample of polymer dissolved in PBS buffer (pH 7.4) was dropped 1.40 m onto a chilled glass coverslip sat on a piece of polished aluminium placed on dry ice. Upon hitting the chilled glass coverslip, a wafer with diameter of approximately 10 mm and thickness 10 μm was formed instantaneously. The glass coverslip was transferred onto the Linkam cryostage and held at −8 °C under N_2_ for 30 minutes. Photographs were obtained using an Olympus CX 41 microscope with a UIS-2 20x/0.45/∞/0-2/FN22 lens and crossed polarizers (Olympus Ltd, Southend on sea, UK), equipped with a Canon DSLR 500D digital camera. Images were taken of the initial wafer (to ensure that a polycrystalline sample had been obtained) and after 30 minutes. Image processing was conducted using Image J, which is freely available^43^. In brief, ten of the largest ice crystals were measured and the single largest length in any axis recorded. This was repeated for at least three wafers and the average (mean) value was calculated to find the largest grain dimension along any axis. The average of this value from three individual wafers was calculated to give the mean largest grain size (MLGS). This average value was then compared to that of a PBS buffer negative control providing a way of quantifying the amount of IRI activity.

## Additional Information

**How to cite this article**: Mitchell, D. E. *et al.* Gold Nanoparticle Aggregation as a Probe of Antifreeze (Glyco) Protein-Inspired Ice Recrystallization Inhibition and Identification of New IRI Active Macromolecules. *Sci. Rep.* **5**, 15716; doi: 10.1038/srep15716 (2015).

## Supplementary Material

Supporting Information
