# Supplementary material for: Gold Nanoparticle Aggregation as a Probe of Antifreeze (Glyco) Protein-Inspired Ice Recrystallization Inhibition and Identification of New IRI Active Macromolecules
Source: Sci Rep. 2015 Oct 26;5:15716. doi: 10.1038/srep15716 (PMC4620503; doi:10.1038/srep15716)
Supplement: Supporting Information [file srep15716-s1.pdf]

Supplementary Information for

Gold Nanoparticle Aggregation as a Probe of Antifreeze (Glyco) Protein-Inspired Ice Recrystallization Inhibition and Identification of New IRI Active Macromolecules

Daniel E. Mitchell, [a,b] Thomas Congdon, [a] Alison Rodger [a] and Matthew I. Gibson [a]*

[a] Department of Chemistry, University of Warwick, Gibbet Hill Road, Coventry, CV4 7AL, UK.

[b] MOAC DTC, University of Warwick, Gibbet Hill Road, Coventry, CV4 7AL, UK.

*Corresponding Author e-mail: m.i.gibson@warwick.ac.uk.

Synthesis and Characterisation of Poly(vinyl alcohol), PVA.

The synthesis of PVA used in this study, was conducted as described in Congdon *et al*[1]. The method is explained below and shown in Figure S1. Table S1 provides characterization details of the polymers selected for this study

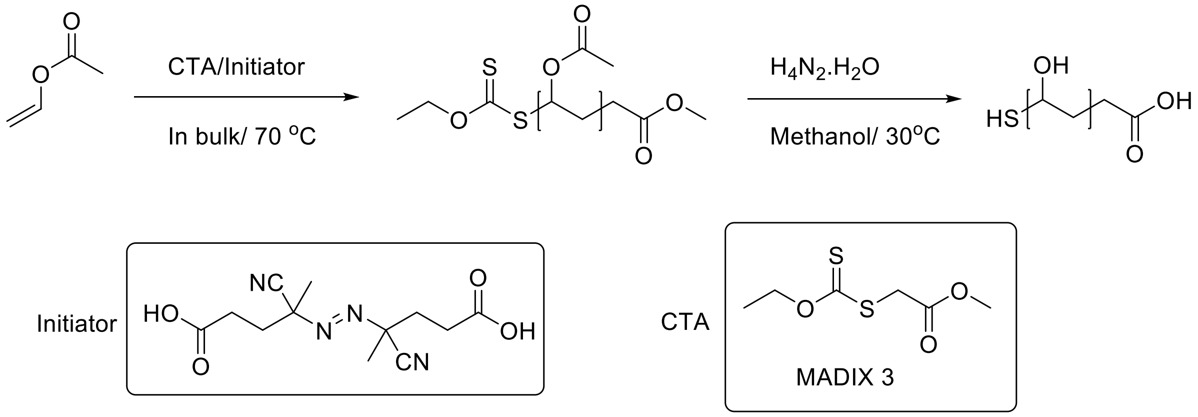

Figure S1. Schematic of the synthesis of PVA using RAFT polymerisation.

**Entry**	**[M]/ [CTA]**$^{(a)}$	**Conv**$^{(b)}$ **%**	$\mathbf{M_{n,theo}}$ $^{(c)}$ **g.mol**$^{-1}$	$\mathbf{M_{n,NMR}}$ $^{(d)}$ **g.mol**$^{-1}$	$\mathbf{M_{n,SEC}}$ **g.mol**$^{-1}$	**Đ** $^{(e)}$	$\mathbf{DP_N}$ $^{(b)}$	**PVA**$^{(g)}$
$\mathbf{PVAc_{10}}$	10	83.2	860	900	870	1.18	10	$\mathbf{PVA_{10}}$
$\mathbf{PVAc_{30}}$	50	60.0	4300	2580	2700	1.45	30	$\mathbf{PVA_{30}}$
$\mathbf{PVAc_{56}}$	100	54.7	8600	4700	5100	1.23	56	$\mathbf{PVA_{56}}$
$\mathbf{PVAc_{154}}$	200	73.8	17200	12700	13800	1.45	154	$\mathbf{PVA_{154}}$
$\mathbf{PVAc_{246}}$	300	80.1	25800	20660	21700	1.39	246	$\mathbf{PVA_{246}}$

Table S1. Details of polymers used in this study. (a) Monomer to RAFT agent ratio; (b) Determined by ^{1}H NMR spectroscopy; (c) Theoretical M_n determined from monomer to RAFT agent ratio; (d) Determined by ^{1}H NMR; (e) Determined by SEC in THF using PMMA standards; (f) Number-average degree of polymerization; (g) Corresponding PVA prepared by hydrolysis of the respective PVAc polymer.

Synthesis of methyl(ethoxycarbonothioyl)sulfanyl acetate (MADIX 2)

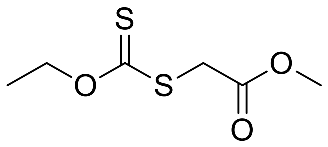

Figure S2. Structure of methyl(ethoxycarbonothioyl)sulfanyl acetate

Ethanol (70 mL) was added to a round bottom flask equipped with a stir bar. Potassium hydroxide (11.45 g, 0.2 mol) was added and left to dissolve for 1 h. Carbon disulphide (12.1 mL, 0.2 mol) was added dropwise, forming a yellow solution, which was left for 5 h. Methyl bromoacetate (6.5 mL, 0.06 mol) was added dropwise and the solution left to stir overnight. The solution was filtered and washed with cold ethanol and concentrated *in vacuo*. The crude product was partitioned in DCM and sat. brine solution and the organic fraction concentrated *in vacuo*. The residue was washed through a column of basic alumina using pure ethyl acetate as the eluent. The fractions were concentrated *in vacuo* and then dried under vacuum. Yield 5.25 g 46%. ^{1}H NMR ($CDCl_3$): δ = 1.42 (2H, t, J=7.2, $CH_3C\textit{H}_2$), 3.76 (3H, s, $C\textit{H}_3O$), 3.92 (2H, d, J=7, $SC\textit{H}_2$), 4.64 (3H, q, J=7.2, $C\textit{H}_3CH_2$). ^{13}C NMR ($CDCl_3$): δ = 14.0 (CH_2–$\textit{C}H_3$), 37.7 ($\textit{C}H_2$), 61.0($CO_2\textit{C}H_3$), 70.4 ($\textit{C}H_2$–CH_3), 167.7 (C=O), 212.4 (C=S).

Polymerisation of Vinyl Acetate Using MADIX 2

As a representative example, MADIX 2 (0.21 g, 0.99 mmol), vinyl acetate (4.67 g, 2.64 mmol), and ACVA (4,4′-azobis(4-cyanovaleric acid); 0.013 g) were added to a stoppered vial. The solution was thoroughly degassed under a flow of N_2 for 20 min, and the reaction mixture was then allowed to polymerize at 68 °C for typically 15 h. The yellow solutions were then cooled to room temperature. Poly(vinyl acetate) was then recovered as a yellow sticky solid after precipitation into hexane. The hexane was then decanted and the poly(vinyl acetate) was redissolved in THF, which was then concentrated *in vacuo* and thoroughly dried under vacuum at 40 °C for 24 h, forming a white solid. Representative characterization data for $PVAc_{56}$: ^{1}H NMR (400 MHz, $CDCl_3$) δ 4.61 (−C*H*O–CH_2, br, 1H), 1.74 (−CO–CH_3, br, 3H), 1.53 (−CH_2–, br, 2H); M_n^{SEC}(THF) = 5100 Da, M_w/M_n = 1.23.

Hydrolysis of Poly(vinyl acetate) to Poly(vinyl alcohol)

As a representative example, poly(vinyl acetate) (1.5 g, 3300 Da, M_n/M_w = 1.22) was dissolved in a methanol (20 mL) and hydrazine hydrate solution (15 mL, 80% in water) in a round-bottom flask. The reaction mixture was stirred at 30 °C for 2 h. The reaction mixture was then dialyzed using distilled water and poly(vinyl alcohol) was recovered as a spongy white solid by freeze-drying the dialysis solution. Deacetylation was determined by ^{1}H NMR. Representative characterization data for PVA_{56}: ^{1}H NMR (400 MHz, $CDCl_3$) δ 4.00 (−C*H*OH–, br, 1H), 1.68–1.60 (−CH_2–, br, 2H).

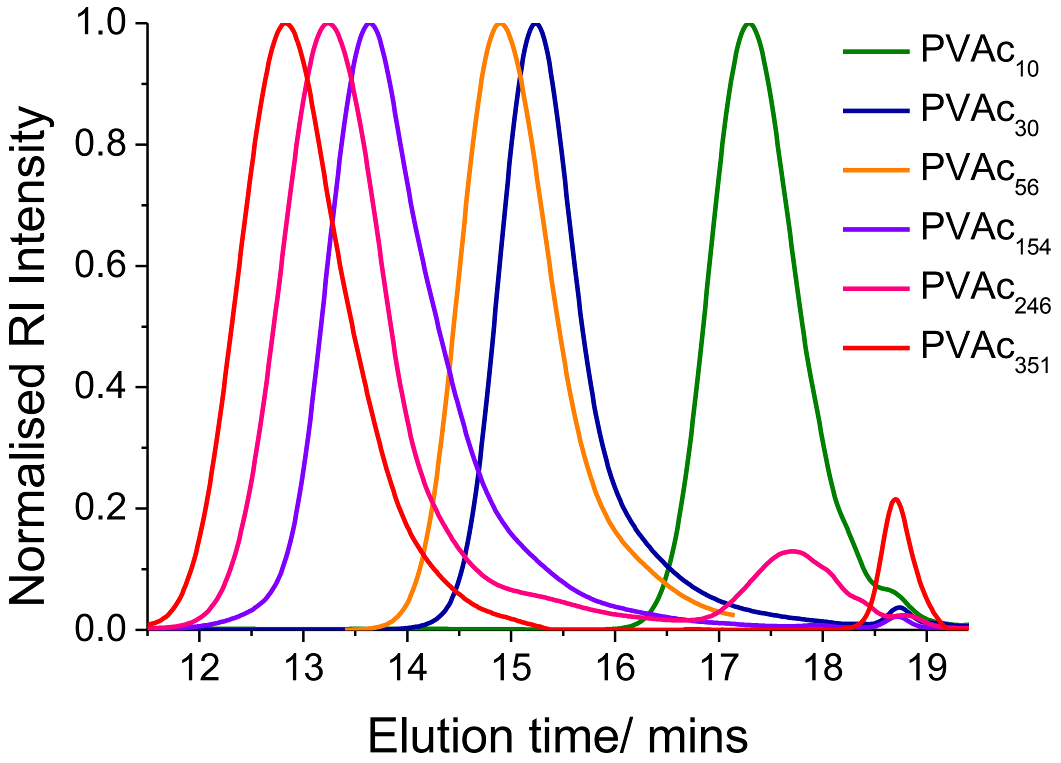

Figure S3. SEC traces of PVA of various molecular weights showing clear differences in size and low polydispersities.

Determining Extent of Aggregation

The change in absorbance at 520 nm was identified as the simplest measurement of aggregation in this study. To account for scattering of aggregated samples, we conveniently measured the difference between the actual absorbance at 520 nm and that of a baseline define between 450 and 680 nm as show in Figure S4.

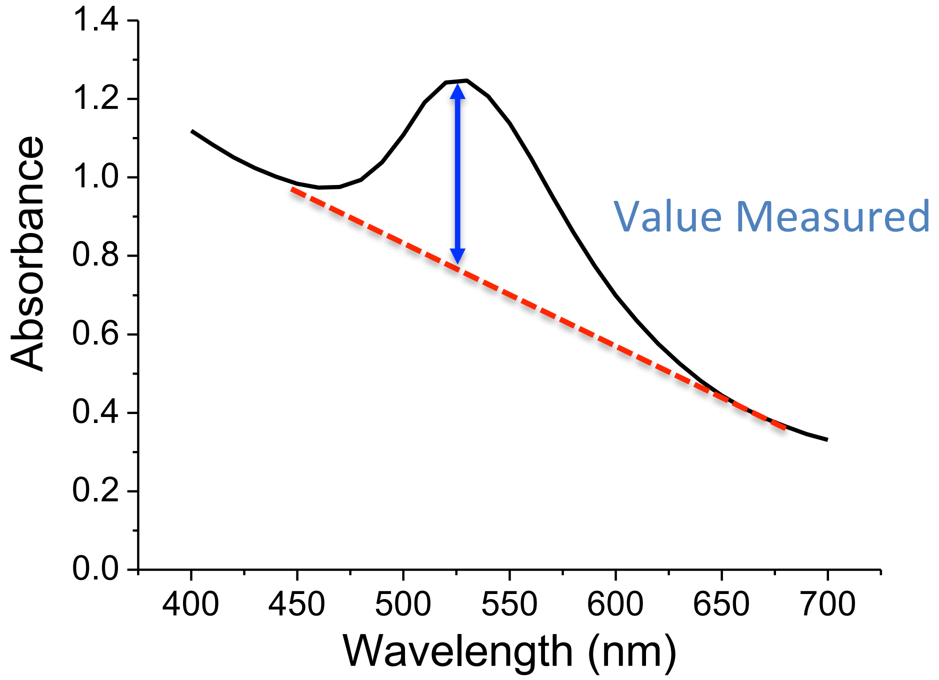

Figure S4. Measurement of absorbance used here.

Variable volume gold particle aggregation experiments

An advantage of the AuNP method is that samples can be tested at a range of different volumes. The rationale for this was that larger volumes would take longer to freeze/thaw, which should increase the amount of ice recrystallization inducing more aggregation and enabling optimization so that there are clear differences between IRI active and non-active compounds. Furthermore, the standard 'splat' assay only uses 10 μL of liquid, which is far from the volumes employed in applications such as cryopreservation, and therefore larger volumes may provide a more predictive test for the additives ultimate application. To this end samples of 50, 100, 200, 500, 1000 and 2000 μL where prepared in 96 and 24 well plates and subjected to the same freeze/thaw cycle as detailed within the paper. The sample depth (which is crucial for freezing rate) verses the volume of the samples is plotted in Figure S4.

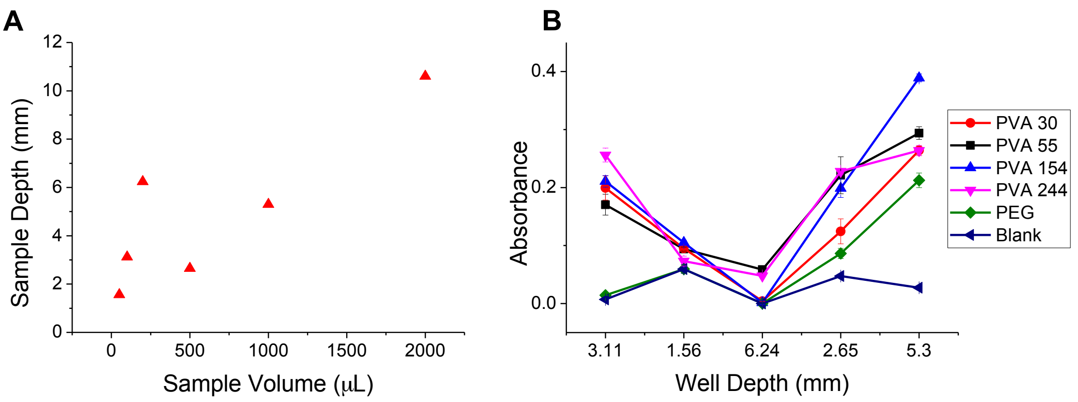

Figure S5. Effect of well depth. (A) Depth of well compared to volume of sample, a 24 well plate is substituted for a 96 well plate for samples volumes greater than 200 μL; (B) Absorbance dependence on the well depth.

From Figure S4, samples with lower well depth provide a generally lower AR (i.e. closer to 1), due to the more rapid thawing that would occur, thus demonstrating that

the thawing rate is important for this assay. Likewise the samples of thicker well depth also seem to show a higher AR possibly due to the depth of sample leading to a greater level of light absorbance. The depth-dependence also suggests that this assay is probing ice recrystallization and not ice shaping, which was previously suggested. Overall the clearest difference between IRI active and no active compounds is found at a well depth of 3.118mm, which corresponds to 100 μL in a 96 well plate. It is therefore recommended that 50 μL of sample to 50 μL of AuNPs making an overall volume of 100 μL be used to investigate the IRI activity of compounds.

Comparison of Absorbance Change to Mean Largest Grain Size for Poly(Amino ethyl methacrylate)-*co*-succinic anhydride

Since PAEMA-*co*-SA has also been shown to exhibit IRI activity the absorbance has been plotted here against mean largest grain size (MLGS) (as a percentage of PBS buffer negative control), Figure S5.

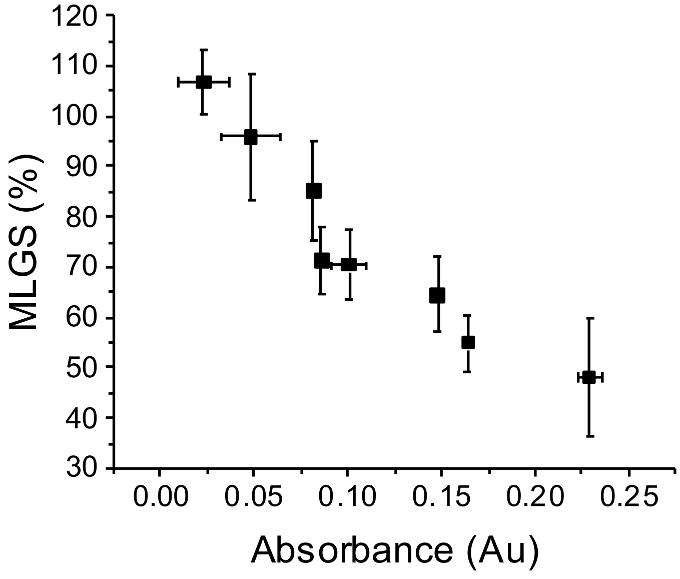

Figure S6. Comparison of PAEMA-*co*-SA polymers mean largest grain size (MLGS) and absorbance at 20mg/ml. MLGS = mean largest grain size relative to a PBS control, expressed as %. Error bars represent ± SD from a minimum of 3 repeats.

Plotting the MLGS absorbance change against each other on the same graph shows a linear correlation and reinforces the premise that the AuNP method is probing the same phenomenon as the "splat test" namely ice recrystallization inhibition.

Concentration Dependence of AuNPs with PVA

To assess how the concentration of AuNPs affects the assay, a serial dilution was performed in the presence of 5mg/ml PVA, figure S6.

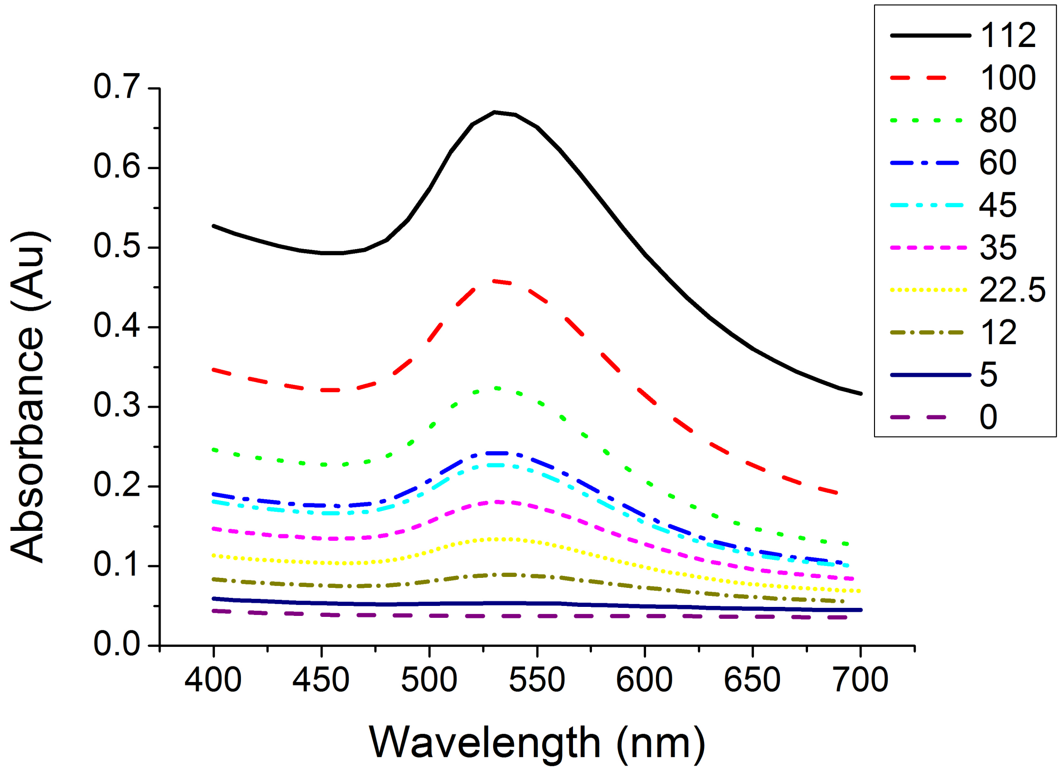

Figure S7. Spectra of AuNPs frozen in the presence of 5mg/ml PVA. Values given in the key are concentration in $\mu g.mL^{-1}$.

From Figure S7 it can be seen there is a reduction in absorbance across the whole spectra as AuNP concentration decreases. However the peak at around 550 nm remains at all concentrations showing that PVA prevents the aggregation of AuNPs. Furthermore if no PVA is added, this 550 nm peak disappears at all concentrations of AuNPs. To maximize the difference between 520 nm and 650 nm a minimum AuNP concentration of 80 μgmL^{-1} is optimal to avoid wastage and still function in the assay.

References.

1. Congdon, T., Notman, R. & Gibson, M.I. Antifreeze (glyco)protein mimetic behavior of poly(vinyl alcohol): detailed structure ice recrystallization inhibition activity study. *Biomacromolecules* 14, 1578-86 (2013).
